# Methods to measure potential spatial access to delivery care in low- and middle-income countries: a case study in rural Ghana

**DOI:** 10.1186/1476-072X-13-25

**Published:** 2014-06-26

**Authors:** Robin C Nesbitt, Sabine Gabrysch, Alexandra Laub, Seyi Soremekun, Alexander Manu, Betty R Kirkwood, Seeba Amenga-Etego, Kenneth Wiru, Bernhard Höfle, Chris Grundy

**Affiliations:** 1 Epidemiology and Biostatistics Unit, Institute of Public Health, Heidelberg University, Heidelberg, Germany; 2 Institute of Geography, Heidelberg University, Heidelberg, Germany; 3 Maternal & Child Health Intervention Research Group, Faculty of Epidemiology and Population Health, London School of Hygiene and Tropical Medicine, London, UK; 4 Kintampo Health Research Center, Ghana Health Service, Kintampo, Ghana; 5 GIScience, Institute of Geography, Heidelberg University, Heidelberg, Germany; 6 Department of Social and Environmental Health Research, London School of Hygiene and Tropical Medicine, London, UK

## Abstract

**Background:**

Access to skilled attendance at childbirth is crucial to reduce maternal and newborn mortality. Several different measures of geographic access are used concurrently in public health research, with the assumption that sophisticated methods are generally better. Most of the evidence for this assumption comes from methodological comparisons in high-income countries. We compare different measures of travel impedance in a case study in Ghana’s Brong Ahafo region to determine if straight-line distance can be an adequate proxy for access to delivery care in certain low- and middle-income country (LMIC) settings.

**Methods:**

We created a geospatial database, mapping population location in both compounds and village centroids, service locations for all health facilities offering delivery care, land-cover and a detailed road network. Six different measures were used to calculate travel impedance to health facilities (straight-line distance, network distance, network travel time and raster travel time, the latter two both mechanized and non-mechanized). The measures were compared using Spearman rank correlation coefficients, absolute differences, and the percentage of the same facilities identified as closest. We used logistic regression with robust standard errors to model the association of the different measures with health facility use for delivery in 9,306 births.

**Results:**

Non-mechanized measures were highly correlated with each other, and identified the same facilities as closest for approximately 80% of villages. Measures calculated from compounds identified the same closest facility as measures from village centroids for over 85% of births. For 90% of births, the aggregation error from using village centroids instead of compound locations was less than 35 minutes and less than 1.12 km. All non-mechanized measures showed an inverse association with facility use of similar magnitude, an approximately 67% reduction in odds of facility delivery per standard deviation increase in each measure (OR = 0.33).

**Conclusion:**

Different data models and population locations produced comparable results in our case study, thus demonstrating that straight-line distance can be reasonably used as a proxy for potential spatial access in certain LMIC settings. The cost of obtaining individually geocoded population location and sophisticated measures of travel impedance should be weighed against the gain in accuracy.

## Introduction

Skilled attendance at birth is recommended to reduce the over 270,000 maternal and three million neonatal deaths that occur annually, most in low-income countries [1]. Many factors influence the use of skilled delivery care, including characteristics of the mother and of the service environment [2]. In many rural high-mortality settings, geographic access to skilled delivery care is poor. While often neglected, the impact of geographic access to skilled care on choice of delivery place is of similar magnitude as that of education or wealth [3].

### Defining access

Access to health care has been conceptualized and operationalized in many ways. Potential and realized access distinguish between stages in the use of care [4]: potential access refers to the availability of services in a geographic area, realized access to the actual use of services after barriers have been overcome. The term “spatial access” encompasses two of the formative five dimensions of access described by Penchansky and Thomas: accessibility, referring to the spatial relationship between location of supply and demand; and availability, referring to the adequacy of provider supply in relation to demand [5,6]. Accessibility and availability are often combined into a single index to measure geographic coverage of care, ranging in complexity from a simple ratio of provider supply to patient demand, to more complex indices such as the two-step floating catchment area method [7,8]. This paper addresses the first dimension, calculating accessibility as in travel impedance, which can be thought of as a measure of the “friction of distance” or the “cost of travel” between locations and expressed in distance or time [9].

### Measures of accessibility in public health research

Distance or travel time between place of residence and a health service location are common measures of travel impedance used in public health research. The simplest approximation is straight-line or Euclidean distance between two points. Geographic information systems (GIS) can be used to model more realistic estimates of travel impedance, such as road network distance or travel time.

In principle, there are two types of data models that can be used to model the cost of travel in GIS separately or in combination: vector data models and raster data models [9]. Vector data models represent traversable paths between points along lines (termed edges) in a network, with anything not on the network being non-traversable ‘empty space’, whereas raster data models represent travel through a pixel (cell) grid, where all space in a defined area is included in the grid. The cost of travel in both model scenarios is determined by an impedance value assigned to each edge in vector models, or each cell, in raster models. Both models can incorporate travel along roads, and raster models usually include topographic features such as land-cover and void areas that cannot be traversed (e.g. lakes, legally restricted areas). Time or season may modify these impedance values, and space-time models can incorporate changes in topography and land-cover as well as population over time.

In practice, the appropriate impedance measure is largely determined by several issues: data availability, geographic context (topography i.e. water bodies and mountains) and cultural context (i.e. common modes of travel). Information on road networks and land-cover is publicly and commercially available in many high-income countries, making more sophisticated estimates of distance and travel time possible (e.g. United States Census Bureau, TIGER/Lines, ESRI). Freely available geographic information mapped by crowdsourcing, such as the OpenStreetMap, can also be used for routing in areas with high quality data [10]. Network models are appropriate in high-income settings like the United States because most travel occurs on roads, whereas in countries with limited infrastructure, travel does not always occur in vehicles or on roads. Additionally, road and land-cover data are not systematically available on a global level, and particularly scarce in low-income countries [11]. Sourcing input data is an important and sometimes difficult task, the quality of the estimates depends on the resolution, accuracy, currency and completeness of the data [12]. Data often come from multiple sources, and it is important to ensure that the different layers of information are temporally coherent, i.e. refer to the same time period. Researchers working in low-income countries often have to spend considerable time and effort to locate data, usually from multiple sources, or digitize road network maps themselves [13].

### Previous studies

Previous comparisons of different measures of geographic accessibility have suggested that the use of Euclidean distance is a poor proxy of access, however, most comparisons have been in high-income countries, and many did not assess the methods against an outcome (Table 1). Two studies conducted in predominantly rural areas in LMICs concluded against Euclidean distance; one in favor of actual travelled distance measured with trackers [14], and one for raster travel time [15]. However, both studies were conducted in more mountainous regions of East Africa, and their findings might not hold true in other flatter settings.

**Table 1:** Studies comparing different methods of calculating travel impedance to health services

**# Ref**	**Author (year) Country**	**Data sources**	**Impedance measures**	**Outcome**	**Comparison method**	**Favoured measure/conclusion**
**Low- and middle-income countries**						
1 [14]	Okwaraji (2012) Ethiopia	1. Geocoded households	1. Euclidean distance	Under 5 child mortality	1. Correlation coefficient	Actual travel distance
		2. Geocoded health center	2. Raster travel time		2. Compare measures of effect	
		3. Land cover, Ethiopia Mapping Agency	3. Actual travel distance			
		4. Digital elevation model from Shuttle Radar Topography Mission (NASA)				
2 [15]	Noor (2006) Kenya	1. Geocoded homesteads	1. Euclidean distance	Predicted specific facility use by febrile children; Proportion of people within one hour of HF	1. Kappa statistic (agreement between predicted and observed facility use)	Raster travel time (transport network model) adjusted for competition
		2. Geocoded HFs	2. Raster travel time (termed transport network model)		2. Linear regression (R^2^)	
		3. Population density at 100 m resolution (Kenya Census 1999)	3. Raster travel time (transport network model), adjusted for competition between facilities		3. Scatter plots	
		4. Road network (Africover, plus manual updates)			4. Spatial mapping	
		5. Topography (Africover, plus updates & Livestock research institute, Nairobi & Park & reserve digital map from Kenya Wildlife Service)				
3 [16]	Costa (2003) Brazil	1. Admissions data from national public health database	1. Euclidean distance	None	1. Maximum difference in distances	“Real” distance
		2. Extracted district of residence from postal codes from national database	2. “Real” distance, estimated as city bus itinerary from district centroid to hospital, adjusted for residence district area			
		3. GIS coordinates for 14 public hospitals				
		4. City transit network map, bus routes				
**High-income countries**						
3 [17]	*Cudnik (2012) USA	1. Patient location via EMS data	1. Euclidean distance	None	1. Wilcoxon signed rank test	Reasonable to use Euclidean distance
		2. HF location via addresses	2. Network distance		2. Spearman rank	
		3. Road network (ArcGIS StreetMap; commercially available)	3. Actual transport distance (in EMS vehicle)		3. Linear regression (R^2^)	
4 [9]	*Delamater (2012) USA	1. Population (US Census 2010)	1. Network travel time	Proportion of state classified as limited access area (LAA)	1. Percentage change in proportion LAA	Depends on research question
		2. Road network (Michigan Center for Geographic Information 2009)	2. Network distance		2. Mapping	
			3. Raster travel time			
			4. Raster distance			
5 [18]	~*Lian (2012) USA	1. Incident breast cancer cases (Missouri cancer registry)	1. Network travel time	Incident odds of late-stage breast cancer	1. Spearman rank	2SFCA
		2. Population coordinates (US Census 2000)	2. Average of 5 shortest network travel times		2. Kappa coefficient	
		3. HF coordinates (FDA)	3. Service density		3. Moran I index	
		4. Road Network (US Census/ TIGER)	4. Two-step floating catchment area (2SFCA)		4. Comparison of effect measures on risk of outcome	
6 [19]	*Jones (2010) USA	1. Population location (Insurance claims data)	1. Euclidean distance	None	1. Wilcoxon’s signed rank sum tests	Network distance
		2. HF location via addresses	2. Network distance		2. Scatter plots	
		3. Road network (no source listed)				
7 [20]	*Apparicio (2008) Canada	1. Population coordinates (Statistics Canada)	1. Euclidean distance	None	1. Spearman rank	Network distance
		2. HF coordinates (Quebec Ministry of Health and Social services)	2. Manhattan distance		2. Absolute differences in measures	
		3. Road network (CanMap street files, commercially available)	3. Network distance		3. Spatial mapping	
			4. Network travel time			
8 [21]	Fone (2006) UK	1. Population via postal survey from Gwent Health Authority	1. Euclidean distance	Perceived accessibility	1. Kruskal-Wallis	Minimal advantage in using sophisticated measures
		2. Population location via census	2. Network travel time		2. Spearman rank	
		3. HF locations from Gwent Health Authority	3. Network distance			
		4. Road network (MapInfo Drivetime software, commercially available)				
9 [22]	Haynes (2006) UK	1. Hospital-based patient questionnaire (with post-codes)	1. Euclidean distance	None	1. Spearman rank	No evidence that GIS estimates better than Euclidean
		2. Geocoded HF location	2. Network travel time		2. Linear regression (R^2^)	
		3. Road network (Ordinance Survey Meridian, digital map)	3. Actual travel time			
10 [23]	*Fortney (2000) USA	1. Population location from previous study sample	1. Euclidean Distance	None (travel time as gold standard)	1. Correlation coefficients	Marginal gains in accuracy using network measures
		2. HF location from physician desk reference database (State licensing board)	2. Network distance		2. Linear regression	
		3. Road network (US Census Bureau)	3. Differences between measures			

Included studies compared Euclidean distance to at least one other method of calculating travel impedance included in our comparison, or compared two other methods used in our comparison (~denotes an exception). Abbreviations: HF = health facility; FDA = US Food and Drug Administration; EMS = emergency medical service; 2SFCA = two-step floating catchment area; LAA = limited access area; NASA = US National Space Agency. *Studies also compared population aggregation methods (e.g. address, census area, census block post/zip-code centroid etc., details not included).

### Study aim and objectives

By comparing different measures of travel impedance, we aim to determine if Euclidean distance can be used as a reasonable proxy for potential spatial access in LMIC settings, using Brong Ahafo region of Ghana as a case study.

This study has three objectives:

1) to investigate the effect of using different geospatial algorithms and data models (vector, raster) on measures of travel impedance (Euclidean distance, network distance, network travel time, raster travel time; Table 2) between population and delivery care,

**Table 2:** Definitions of different impedance measures

**Impedance measure**	**Units**	**Definition**	**Data type**	**GIS tool**
Euclidean distance	Km	Straight-line distance from population to closest health facility	Vector	Near
Network distance	Km	Distance along road network from population to closest health facility, plus Euclidean distance to the road network from the population, and from the road network to the health facility	Vector	Network analyst closest facility
				+ Near
Mechanized network time	Hour	Distance along road network from population to closest health facility multiplied by driving speed on roads, plus Euclidean distance multiplied by off-road walking speed (2 km/h) to the road network from the population, and from the road network to the health facility	Vector	Network analyst closest facility
				+ Near
Non-mechanized network time	Hour	Distance along road network from population to closest health facility multiplied by walking speed on roads (4 km/h), plus Euclidean distance multiplied by off-road walking speed (2 km/h) to the road network from the population, and from the road network to the health facility	Vector	Network analyst closest facility
				+ Near
Mechanized raster time	Hour	Travel time from population to closest health facility, assuming mechanized travel on roads and non-mechanized travel off road according to land cover speeds*, 200 m x 200 m grid	Raster	Least-cost path
Non-mechanized raster time	Hour	Travel time from population to closest health facility, assuming non-mechanized travel on roads (4 km/h) and off roads according to land cover speeds*, 200 m x 200 m grid	Raster	Least-cost path

*GlobCover 2009 [24], GEM European Commission project [25].

2) to assess the potential spatial aggregation error associated with using average population location (village centroid) compared with individually geocoded location (compound of residence) on measures of travel impedance,

3) to compare the association that different proxies for spatial access to care show with facility delivery, i.e. whether or not women use a facility for delivery as a binary outcome variable, using surveillance data over a one-year period from the study area.

## Methods

### Overview & data sources

Ghana is a West African country with a high maternal mortality ratio estimated at 328 per 100,000 in 2011 [26]. The study area consists of 7 contiguous districts with a population of more than 100,000 women of reproductive age (14-45 yrs), where demographic surveillance was established for several field trials [27-29]. Travel occurs on roads, and mainly on foot to the closest health facility, as reported by approximately 58% of households in a 2003 national survey [30].

**Figure 1:**
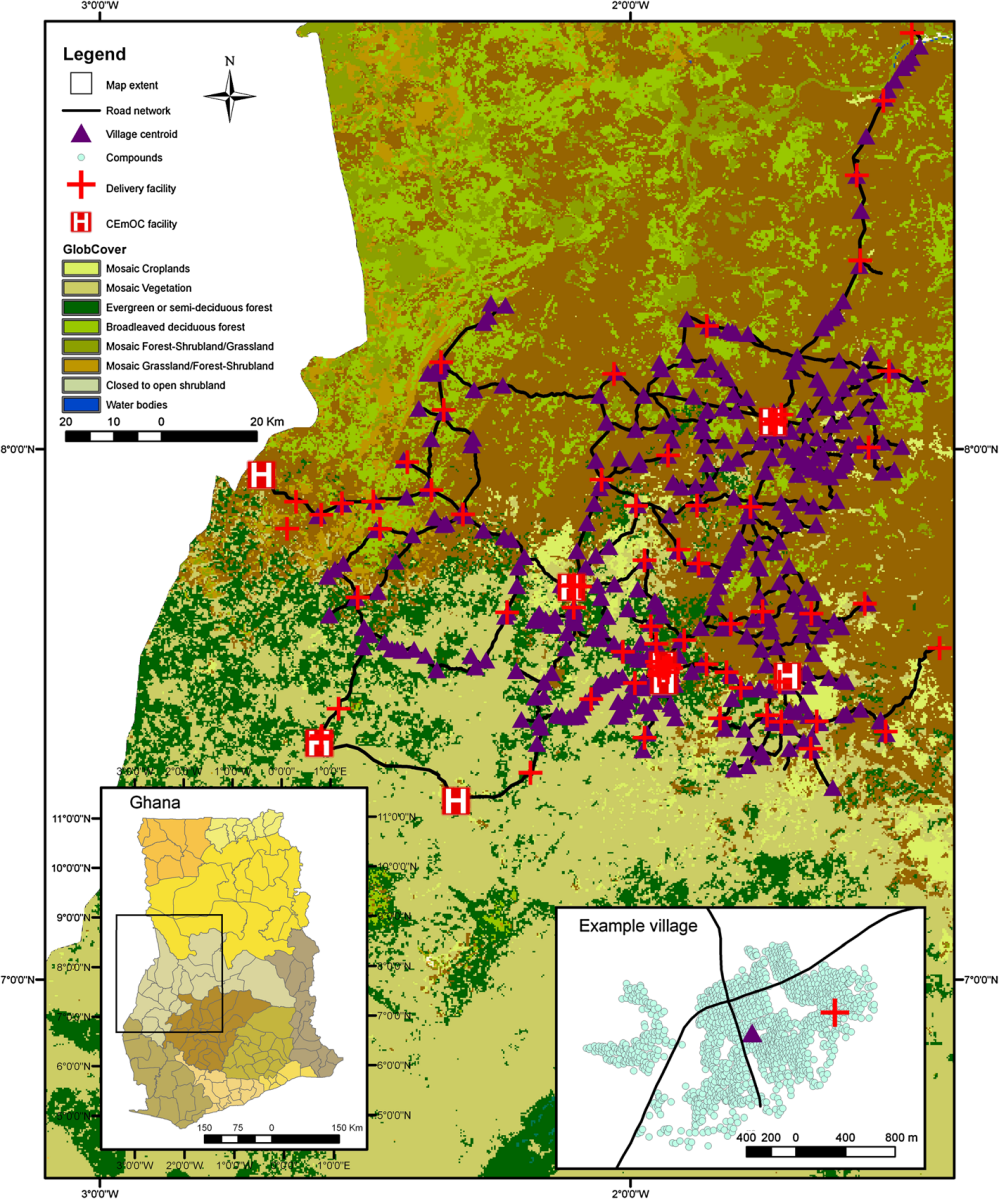
**Study area showing topographic cover in Brong Ahafo region, Ghana.** First inset shows study area in Ghana with administrative divisions. Second inset shows detail of example village with centroid, compounds, road network and a delivery facility.

**Figure 2:**
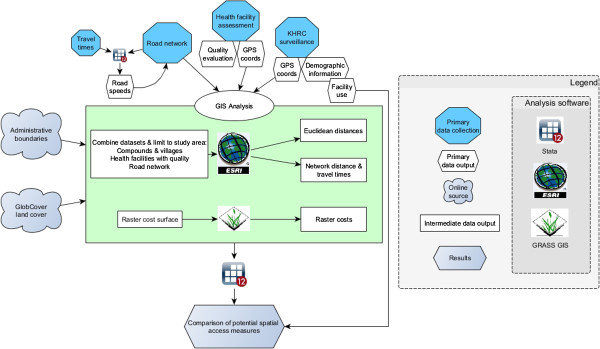
Workflow for geospatial analysis.

A geospatial database of the study area was created, mapping population location in compounds and village centroids, service locations for all health facilities offering delivery care (including higher level facilities with capacity for surgery), and a detailed road network (Figure 1). We included administrative boundaries and topography (land-cover, including water bodies) [24,25]. We combined data sources in a workflow (Figure 2), and describe the fieldwork in more detail here:

1. Road network

A detailed road network of all roads in the study area was created using GPS trackers. The study area covers approximately 15,302 km^2^ and our road network includes over 1,900 km of roads. Extensive deskwork was done in order to transform these road tracks into a network dataset appropriate for analysis in ArcGIS, ensuring functional connectivity between roads. A tool was developed in a PostGIS geodatabase to validate the connectivity of the network roads, and the road network was subsequently cleaned in GRASS GIS [31]. The road network was then integrated into the land-cover raster layer for analysis using a 200 m resolution. Additional information on road condition, surface type, and usability in the rainy season was collected for all roads.

Travel times by vehicle between village centroids were collected for one study district. A total of 88 journey segments were used in order to calibrate road speeds, which were assigned with reference to speeds used in the literature [32,33]. Road speeds ranged from 30 km/h on dirt roads, to 90 km/h on good tarmac roads. Very few roads (four in the study area) were reported as impassable during the rainy season, so we model the dry season scenario only.

2. Health facility census

We conducted a health facility assessment of all 86 geocoded health facilities in the study area to categorize facilities according to the availability and quality of maternal and newborn services: 64 facilities offered delivery services and 8 offered comprehensive emergency obstetric care (CEmOC), i.e. higher-level facilities with the capacity for cesarean section and blood transfusion [34-36]. The majority of the hospitals, health centers, and clinics with delivery care are publically owned, and all maternity homes are operated privately by the Ghana Registered Midwives Association.

3. Surveillance

Surveillance of all women of reproductive age in the study area through monthly visits was undertaken as part of health and demographic surveillance for several field studies [27,28]. The surveillance included taking GPS coordinates of 433 village centroids and, in 173 larger villages, coordinates of 47,537 individual compounds (with a median of 450 compounds per village (IQR 258–844, max 3,204)).

For the analysis of facility use (objective 3), we included villages and compounds where deliveries occurred in 2009 with known birthplace and compound coordinates, resulting in 169 villages, 8,120 compounds and 9,306 births. There was a median of 96 births per village (range 1–634), and a median of 1 birth per compound (range 1–8).

### Distance measures

All six impedance measures were calculated to two levels of care, distance to closest facility with delivery care, and distance to closest facility with CEmOC (Table 2).

In ArcMap version 10.0, we used the Spatial Analyst tool “Near” to calculate Euclidean distances and the Network Analyst tool “Closest Facility Analyst” to calculate network distance and time (ESRI software, California). For the raster-based analyses, we used the cost surface algorithm in GRASS GIS to determine the fastest route (least-cost path) from starting points to given destinations [31,37].

### Analysis

To address objective 1, we used Spearman rank correlation coefficients to compare the six impedance measures within each origin destination pair (i.e. village centroid to closest delivery facility and village centroid to closest CEmOC in a dataset of all villages; compound to closest delivery facility and compound to closest CEmOC in a dataset of all compounds; Table 2).

We assessed potential spatial aggregation error (objective 2) in three ways using the surveillance dataset. First, we compared the correlation of the measures calculated from the two origins, and then whether the different measures identified the same facility as closest from both origins for each birth. Finally, we calculated distance deviance, the absolute difference in distance or time between measures starting from compounds compared to measures starting from village centroid for each birth. These absolute differences represent the potential error in access estimates that result from using average village centroids as opposed to individual compound coordinates, and are dependent on the dispersion of villages.

Spatial access to health care is known to be a facilitator of delivery in a health facility [3]. The impedance measure that is the best proxy of spatial access to delivery care, i.e. has the least measurement error, should then show the strongest association with facility delivery in a regression model (objective 3). We modeled the association of each impedance measure with whether or not a woman delivered in a facility as a binary outcome variable, in a logistic regression model for all births in the study area. For ease of comparison between measures with units in distance and time, we standardized our impedance measures to have a mean approximately equal to zero and standard deviation (SD) of one. In order to account for clustering of women by village, we used logistic regression models with robust standard errors. All analyses were done in Stata version 12.0.

### Ethical considerations

This study uses data collected for the Newhints trial, which was approved by the ethics committees of the Ghana Health Service, Kintampo Health Research Center and the London School of Hygiene and Tropical Medicine (LSHTM) [28]. The additional analyses were approved in an amendment by the LSHTM ethics committee.

## Results

### Different measures of travel impedance

Median Euclidean distance from compounds to closest health facility was less than 1 km, and less than 10 km to the closest CEmOC facility (Table 3). Network distances were longer than Euclidean distances; partly because this measure includes distances to and from the road network (Table 3). Median mechanized network time (i.e. drive time) from compounds to closest facility was 13 minutes (0.22 hours), and to closest CEmOC was 23 minutes. Raster methods produced longer travel times than the network method; this is likely due to the use of the GlobCover topography map, which has higher impedance values (slower speeds) for off-road travel than the network model where we used a fixed speed of 2 km/h.

**Table 3:** Impedance measures from compound and village to closest facility using six methods

	**Compound as origin, n = 47, 537**			**Village as origin, n = 433**		
**Distance to closest health facility**	**Mean (SD)**	**Median (IQR)**	**Range**	**Mean (SD)**	**Median (IQR)**	**Range**
Euclidean (km)	3.01 (4.47)	0.91 (0.49-4.16)	0.0038 - 23.88	6.19 (4.59)	5.74 (2.18-9.12)	0.026 - 23.44
Network distance(km)	3.91 (5.33)	1.47 (0.85-4.99)	0.018 - 35.33	8.15 (6.53)	7.31 (2.86-11.78)	0.036 - 40.99
Mechanized network time (hr)	0.26 (0.17)	0.22 (0.12-0.35)	0.0055 - 1.11	0.26 (0.18)	0.23 (0.12-0.35)	0.008 - 1.00
Non-mechanized network time (hr)	1.08 (1.32)	0.50 (0.29-1.31)	0.0085 - 8.90	2.09 (1.63)	1.89 (0.77-3.00)	0.013 - 10.26
Mechanized raster time (hr)	0.31 (0.27)	0.25 (0.14-0.40)	0 - 2.78	0.27 (0.27)	0.23 (0.13-0.32)	0 - 2.40
Non-mechanized raster time (hr)	1.20 (1.40)	0.67 (0.31-1.50)	0 - 9.16	2.18 (1.64)	1.97 (0.88-3.06)	0 - 9.81
**Distance to CEmOC**	**Mean (SD)**	**Median (IQR)**	**Range**	**Mean (SD)**	**Median (IQR)**	**Range**
Euclidean (km)	12.53 (14.1)	9.64 (1.49-17.18)	0.028 - 84.27	17.36 (13.45)	14.73 (8.57-23.31)	0.15 - 84.00
Network distance (km)	15.08 (16.14)	11.25 (2.32-21.76)	0.041 - 90.63	22.18 (17.11)	19.38 (10.86-28.66)	0.53 - 90.27
Mechanized network time (hr)	0.42 (0.25)	0.39 (0.23-0.57)	0.0055 - 1.40	0.50 (0.27)	0.45 (0.29-0.66)	0.016 - 1.35
Non-mechanized network time (hr)	3.87 (4.02)	2.95 (0.72-5.50)	0.017 - 22.76	5.61 (4.26)	4.93 (2.74-7.20)	0.13 - 22.59
Mechanized raster time (hr)	0.49 (0.35)	0.44 (0.23-0.68)	0 - 2.89	0.52 (0.34)	0.46 (0.27-0.68)	0.009 - 2.40
Non-mechanized raster time (hr)	4.05 (1.14)	3.18 (0.90-5.75)	0 - 23.79	5.78 (4.37)	5.01 (2.76-7.45)	0.10 - 23.26

Note: n = 47,537 compounds in 173 villages for compound calculations, n = 433 villages for village calculations, compound and village statistics should not be compared due to different sample sizes. n = 64 delivery facilities; n = 8 CEmOC facilities.

Median Euclidean distance from villages as origin to the closest delivery facility was 5.7 km; median distance to the closest delivery facility was less than 1 km from compounds (Table 3). However, the median of all villages should not be compared to the median of all compounds, as there are many more compounds than villages, and larger villages with many compounds are more likely to also have a health facility, leading to a shorter median distance and travel time for measures from compound. A fair comparison of measures from compound to measures from village is made in the surveillance dataset of births from the 169 villages with both coordinates (see below, spatial aggregation error).

Distances to the closest CEmOC facility were over 10 km longer and mechanized travel times 2–2.5 times longer than to the closest delivery facility. For instance, median network distance from village to closest CEmOC was 19.4 km compared to 7.3 km to closest delivery facility. Non-mechanized travel times (i.e. walking) were much longer to CEmOC facilities than to delivery facilities in general: walking from one’s compound along the road network to the closest delivery facility would take a median of 30 minutes, whereas walking to the closest CEmOC facility would take nearly 3 hours (Table 3).

With the exception of the mechanized measures, Spearman coefficients showed that distance and travel time measures were highly correlated with each other (r > 0.89, Tables 4 & 5). Correlation between travel times incorporating mechanized travel (i.e. driving) with the other measures was low; Euclidean distance and mechanized raster time from compound to closest delivery facility were the least correlated (r = 0.39). The highest correlation was between network distance and network walking time from village centroid to the closet CEmOC facility (r = 0.99). Correlations between measures showed a similar pattern from both origins (compounds and villages), with slightly higher correlations for the longer distances to CEmOC facilities.

**Table 4:** Spearman rank correlation coefficients (r) between different impedance measures and same health facility identified as closest using different impedance measures (%) for impedance measures calculated to closest delivery facility

	**Euclidean distance**		**Network distance**		**Mechanized network time**		**Non-mechanized network time**		**Mechanized raster time**	
	**r**	**%**	**r**	**%**	**r**	**%**	**r**	**%**	**r**	**%**
**Compound as origin**^**1**^										
Network distance (km)	0.9330	91.6	1							
Mechanized network time (hr)	0.3904	89.6	0.5485	97.0	1					
Non-mechanized network time (hr)	0.8921	91.6	0.9824	100	0.6681	97.0	1			
Mechanized raster time (hr)	0.3785	72.9	0.4519	68.7	0.7226	67.8	0.5335	68.7	1	
Non-mechanized raster time (hr)	0.8763	91.0	0.8978	90.7	0.6649	88.5	0.9278	90.7	0.6748	73.6
**Village as origin**^**2**^										
Network distance (km)	0.9584	80.4	1							
Mechanized network time (hr)	0.5885	78.8	0.6842	98.5	1					
Non-mechanized network time (hr)	0.9529	80.4	0.9983	100	0.7177	96.5	1			
Mechanized raster time (hr)	0.5804	64.9	0.6389	72.5	0.7964	73.4	0.6616	72.5	1	
Non-mechanized raster time (hr)	0.9404	80.6	0.9813	91.0	0.7497	89.8	0.9876	91.0	0.7247	76.2

^1^n = 47,537 compounds ^2^n = 433 villages; n = 64 delivery facilities.

**Table 5:** Spearman rank correlation coefficients (r) between different impedance measures and same health facility identified as closest using different impedance measures (%) for impedance measures calculated to closest CEmOC facility

	**Euclidean distance**		**Network distance**		**Mechanized network time**		**Non-mechanized network time**		**Mechanized raster time**	
	**r**	**%**	**r**	**%**	**r**	**%**	**r**	**%**	**r**	**%**
**Compound as origin**^**1**^										
Network distance (km)	0.9849	91.6	1							
Mechanized network time (hr)	0.7388	89.4	0.7852	91.0	1					
Non-mechanized network time (hr)	0.9805	91.6	0.9980	100	0.8116	97.0	1			
Mechanized raster time (hr)	0.5333	68.5	0.5847	73.4	0.8868	67.8	0.6171	68.7	1	
Non-mechanized raster time (hr)	0.9680	90.4	0.9852	97.8	0.8439	88.5	0.9923	90.7	0.6749	73.6
**Village as origin**^**2**^										
Network distance (km)	0.9714	84.8	1							
Mechanized network time (hr)	0.7115	87.3	0.7576	87.5	1					
Non-mechanized network time (hr)	0.9707	84.8	0.9996	100	0.7703	87.5	1			
Mechanized raster time (hr)	0.6287	65.6	0.6794	65.4	0.9417	75.5	0.6931	65.4	1	
Non-mechanized raster time (hr)	0.9703	84.1	0.9949	98.6	0.7905	87.1	0.9966	98.6	0.7232	65.8

^1^n = 47,537 compounds ^2^n = 433 villages; n = 8 CEmOC facilities.

Euclidean distance identified the same closest delivery facility as the other measures, except mechanized raster time, for about 80% of village centroids and about 90% of compounds (Table 4). The three network-based measures identified the same closest delivery facility for over 97% of the villages and compounds. Mechanized raster time differed most, identifying the same closest facility for the fewest villages and compounds as other methods.

### Spatial aggregation error

We assessed the influence of spatial aggregation, i.e. how using village centroids (average compound location) differs from using individual population location (compound coordinates) when calculating distance and travel time to health facilities using the surveillance dataset (9,306 births). Correlation coefficients between the two options were high for most measures (r >0.82, Table 6). The same delivery facility was identified as closest in over 85% of births and the same CEmOC facility in over 97% of births (Table 6).

**Table 6:** Spearman rank correlation coefficients (r) and proportion of facilities identified as closest (%) between measures calculated from compound compared to measures calculated from village centroid, n = 9,306 births

**Distance measure**	**Closest delivery facility**		**Closest CEmOC facility**	
	**r**	**%**	**r**	**%**
Euclidean (km)	0.8296	87.8	0.9647	97.7
Network distance (km)	0.8263	88.6	0.9680	98.7
Mechanized network time (hr)	0.7071	86.2	0.8512	97.5
Non-mechanized network time (hr)	0.8324	88.6	0.9708	98.7
Mechanized raster time (hr)	0.5243	86.7	0.6580	97.4
Non-mechanized raster time (hr)	0.8377	85.7	0.9672	98.7

The mean absolute distance deviance (comparing distances from compounds and villages) for the Euclidean measure was 250 m to closest delivery facility and 300 m to closest CEmOC facility (Table 7). There was a larger difference in network distance estimates, 380 m to closest delivery facility and 460 m to closest CEmOC facility. The non-mechanized raster time measure showed the largest difference in time estimates, with a 12 minute difference to the closest delivery facility, and a 14 minute difference to CEmOC. For 90% of the births in the surveillance dataset, the deviance between measures calculated from village and compound was less than 30 minutes for any of the time measures to closest delivery facility, and less than 35 minutes to the closest CEmOC facility. The means and standard deviations of the impedance values calculated from village centroid and compound in the surveillance dataset were almost exactly the same (Table 8).

**Table 7:** Absolute difference in measures to closest delivery facility calculated from compound compared to measures calculated from village centroid, n = 9,306 births

**Distance to closest delivery facility**^**1**^	**Mean (SD)**	**Median (IQR)**	**90%**	**95%**	**Range**
Euclidean (km)	0.25 (0.24)	0.18 (0.08-0.34)	0.57	0.74	8.34E-06 - 2.64
Network distance (km)	0.38 (0.35)	0.28 (0.13-0.52)	0.84	1.11	0.000098 - 2.87
Mechanized network time (hr)	0.092 (0.10)	0.057 (0.025-0.12)	0.22	0.31	5.66E-07 - 0.74
Non-mechanized network time (hr)	0.13 (0.12)	0.09 (0.041-0.17)	0.30	0.39	0.000041 - 0.85
Mechanized raster time (hr)	0.17 (0.23)	0.16 (0.0042-0.18)	0.46	0.57	0 - 2.22
Non-mechanized raster time (hr)	0.20 (0.25)	0.13 (0.50-0.24)	0.48	0.69	0 - 2.42
**Distance to closest CEmOC**^**2**^	**Mean (SD)**	**Median (IQR)**	**90%**	**95%**	**Range**
Euclidean (km)	0.30 (0.31)	0.20 (0.09-0.41)	0.69	0.96	0.000094 - 2.94
Network distance (km)	0.46 (0.48)	0.30 (0.13-0.63)	1.12	1.48	9.54E-06 - 3.21
Mechanized network time (hr)	0.083 (0.089)	0.055 (0.024-0.11)	0.20	0.26	8.94E-07 - 0.69
Non-mechanized network time (hr)	0.14 (0.14)	0.10 (0.013-0.19)	0.35	0.46	3.48E-05 - 0.94
Mechanized raster time (hr)	0.18 (0.25)	0.16 (0.005-0.18)	0.50	0.68	0 - 2.22
Non-mechanized raster time (hr)	0.23 (0.27)	0.15 (0.050-0.30)	0.58	0.79	0 - 2.04

^1^n=64 delivery facilities; ^2^n=8 CEmOC facilities.

**Table 8:** Mean, standard deviation and effect of measures to closest facility on use of facility for delivery, n = 9,306 births

**Distance to closest delivery facility**	**Compound as origin**			**Village centroid as origin**		
	**Mean (SD)**	**OR (95% CI)**	**p-value**	**Mean (SD)**	**OR (95% CI)**	**p-value**
Euclidean (km)	3.09 (4.67)	0.33 (0.27-0.40)	<0.001	3.09 (4.68)	0.33 (0.27-0.40)	<0.001
Network distance(km)	3.85 (5.39)	0.33 (0.26-0.42)	<0.001	3.73 (5.40)	0.33 (0.26-0.43)	<0.001
Mechanized network time (hr)	0.25 (0.17)	0.74 (0.55-0.98)	0.038	0.20 (0.17)	0.84 (0.60-1.17)	0.30
Non-mechanized network time (hr)	1.06 (1.34)	0.33 (0.26-0.43)	<0.001	1.01 (1.34)	0.34 (0.27-0.43)	<0.001
Mechanized raster time (hr)	0.28 (0.26)	0.71 (0.58-0.87)	0.001	0.23 (0.26)	0.91 (0.65-1.27)	0.57
Non-mechanized raster time (hr)	1.16 (1.43)	0.33 (0.26-0.43)	<0.001	1.12 (1.42)	0.53 (0.28-0.45)	<0.001
**Distance to closest CEmOC**	**Mean (SD)**	**OR (95% CI)**	**p-value**	**Mean (SD)**	**OR (95% CI)**	**p-value**
Euclidean (km)	12.40 (15.05)	0.41 (0.33-0.50)	<0.001	12.34 (15.03)	0.41 (0.33-0.50)	<0.001
Network distance (km)	14.94 (17.25)	0.45 (0.36-0.56)	<0.001	14.81 (17.20)	0.45 (0.36-0.56)	<0.001
Mechanized network time (hr)	0.41 (0.26)	0.50 (0.39-0.63)	<0.001	0.37 (0.24)	0.53 (0.42-0.68)	<0.001
Non-mechanized network time (hr)	3.83 (4.30)	0.45 (0.53-0.83)	<0.001	3.78 (4.28)	0.45 (0.36-0.46)	<0.001
Mechanized raster time (hr)	0.47 (0.35)	0.66 (0.32-0.72)	<0.001	0.40 (0.32)	0.76 (0.54-1.08)	0.122
Non-mechanized raster time (hr)	4.00 (4.43)	0.45 (0.36-0.46)	<0.001	3.93 (4.38)	0.46 (0.37-0.57)	<0.001

Note: all measures are standardized to mean = (approx.) 0 & standard deviation = 1.

### Association with facility use

We modeled the association between each impedance measure and facility delivery as a binary outcome, with the assumption that the best proxy for access to care would show the strongest association with use of a facility for delivery. Effect estimates (odds ratios) for facility use were the same for all non-mechanized impedance measures from compound to closest delivery facility (Table 8): the odds of women delivering in a health facility decreased by 67% per standard deviation (SD) increase in each measure to closest delivery facility (OR = 0.33). When calculated from village centroid, the effect of non-mechanized network and raster time was slightly smaller than when calculated from compound as origin. There was less evidence of an association and a smaller effect with mechanized measures from both origins (e.g. OR 0.91; 95%CI 0.65-1.27; p = 0.569 for mechanized raster time from village centroid; Table 8).

We modeled access to any delivery facility and access to CEmOC separately because of the longer distances to CEmOC facilities, and found that effect estimates followed a similar pattern for both facility types. All non-mechanized measures suggest an approximately 55% decrease in odds of delivering in a health facility per SD increase in measure to the closest CEmOC (from both origins), with Euclidean distance showing the largest effect (OR = 0.41 95%CI 0.33-0.50, Table 8). Again, time measures incorporating driving showed a smaller effect.

As a sensitivity analysis, we also modeled the association of these impedance measures with facility delivery using quintiles, log-transformed and binarized measures, as well as adjusting for several potential confounders (age, parity, wealth quintile) with similar results (data not shown).

## Discussion

We comprehensively compared six commonly used measures of travel impedance in a predominantly rural area in Ghana’s Brong Ahafo region as a case study for access measures in certain LMIC settings. We estimated the potential spatial aggregation error using average population location (village centroid) compared with individually geocoded location (compound). We assessed each measure as a proxy for potential spatial access, modeling the association between facility use and travel impedance measures to all delivery facilities and to CEmOC facilities, separately. We showed that measures calculated with different methods were highly correlated with each other, and identified the same facilities as closest for over 80% of villages, with the exception of mechanized cost. Measures calculated from individually geocoded locations (compounds) were highly correlated with measures calculated from village centroids, and identified the same facility as closest for over 85% of births and the same CEmOC facility as closest for approximately 98% of births in the surveillance data. Higher travel impedance was associated with lower facility use for delivery, and this association was of similar magnitude for all travel impedance measures except for mechanized travel.

In contrast to our results, two previous comparisons of distance measures in predominantly rural LMICs favored sophisticated measures over Euclidean distance. A study in Kenya compared models predicting use of specific facilities by febrile children, and found that the Euclidean distance model was less accurate than models using raster measures (called “transport network models” because they incorporated the transport network) [15]. However, the predictive accuracy of the Euclidean and unadjusted raster models was similar (kappa Euclidean 0.71 vs. kappa unadjusted raster 0.73); they differed more substantially when the raster model was adjusted for competition between facilities (kappa 0.83). This suggests it could have been the adjustment for competition in facility types that increased the accuracy of the raster model rather than the access measure itself. In a study region in Ethiopia described as mountainous with a poor road network and difficult terrain, no association was found between under-five mortality and straight-line distance (p value = 0.398), compared to a strong association with actual distance travelled (p value = 0.016) [14]. As we found that Euclidean distance performed as well as the other measures in predicting the odds of facility use, we cannot conclude that it is always better to use sophisticated measures, but that it rather depends on context.

Travel time is determined by many factors in addition to distance, including mode of travel. Incorporating mechanized modes of transport into travel time measures makes assumptions about access to and use of motorized vehicles. For our travel time estimates, speeds were determined empirically for roads, and obtained from a globally available topographic map for land-cover. We assumed the same travel speeds for the entire population, and our estimates should be interpreted as an average estimate for the population as a whole. Assuming that access is a good predictor of facility use, the weaker associations between facility use and driving times indicate that either our road speeds were inaccurate, or that women in our study area do not travel by vehicle to delivery facilities. The reality of an individual woman’s journey to a health facility may include multiple transport modes, such as a combination of walking, public transportation, and hiring taxis, which we were unable to fully take into account due to lack of information on individual’s travel modes.

Obtaining individually geocoded population locations is difficult due to issues of privacy and anonymity and in high-income countries accessibility is usually measured from aggregate locations, such as census tracts or zip codes. Aggregation error arises from the distribution of individuals in a spatial unit, and could affect associations with health outcomes measured on an individual level [20,38]. Results and interpretations vary widely in studies evaluating aggregation errors, and there does not seem to be a generally agreed upon cutoff for this error [38]. Authors of a study in the US comparing individual addresses to zip-code centroids conclude that a deviance <12 miles (19 km) for 95% of the population is not appreciably large [19], while a study in Canada found a deviance <1.5 km for 95% of the population, and considered the >1.5 km deviance for the remaining 5% a significant error [39]. Like other studies in Africa [13], we were able to collect individually geocoded locations and compared these to an aggregate measure, village centroid. While there were some extreme deviances (up to 3.2 km and 2.4 hours), the magnitude of the median deviance (e.g. 180 m Euclidean) was much smaller than the median value of the measures themselves (e.g. 910 m Euclidean). However, a different facility was identified as the closest using different origins for approximately 13% of the births in our surveillance dataset, which may have implications for access if these facilities offer different levels or types of care. The magnitude of this error obviously depends on the size of villages and the dispersion of compounds within villages, which varies with context. Deciding whether village centroids are a sufficient proxy for population location should be made based on the study area context as well as the research question.

As we do not have a gold standard with which to compare the various travel impedance measures to identify the “best” impedance measure, deciding which to use in an analysis is a matter of appropriateness and accuracy which can differ depending on the purpose of the analysis [39]. As all of the non-mechanized measures we included showed almost exactly the same association with facility use from both origins, we conclude that in similarly flat LMIC contexts, simple methods to calculate travel impedance can be reasonably used as proxies for potential spatial access. Euclidean distance is an accurate measure of the distance between two points and does not necessitate making any assumptions regarding mode of travel. For the purpose of comparing relative access to care as opposed to describing detailed journey paths, obtaining accurate estimates may be more important than aspiring to realism.

## Conclusion

Apprehension towards using Euclidean distance as a proxy for access, and the high cost (in time and money) of investing in sophisticated GIS techniques should not be a deterrent to research on access to care in rural low-income settings. Where poor access to care is a major barrier to health, this missing evidence may have real consequences for policy and populations. Using a case study in rural Ghana, we have shown that Euclidean distances from village centroids can be used as a reasonable proxy for individual potential spatial access, which can be generalized to other similar topographic and cultural contexts (i.e. non-mountainous regions, without major water bodies). The accuracy of sophisticated impedance measures depends on the quality of the input data and validity of assumptions regarding travel mode, and the possibility for error in these assumptions may decrease their usefulness. We suggest that for researchers faced with scarce and disparate data sources in relatively flat low-income countries, the use of Euclidean distance from an aggregate measure of population location is an acceptable proxy for access. Furthermore, we suggest that the extra time and effort required for sophisticated and individually geo-referenced methods are justifiable only when a high level of accuracy and completeness of the input data can be assured.

## Competing interests

The authors declare that they have no competing interests.

## Authors’ contributions

RN processed and prepared the geospatial database for analyses, calculated the Euclidean and network based impedance measures, designed and carried out the statistical analyses and drafted the manuscript. SG developed the research question, supervised the statistical analyses and contributed to writing the manuscript. AL and BH calculated the raster based impedance measures, contributed to the design of the statistical analyses and contributed to the writing of the manuscript. CG supervised and coordinated the GIS data collection and developed the research question. SAE supervised and coordinated the GIS data collection. KW implemented the GIS data collection and supported the preparation of the geospatial database. SS, AM and BRK (with others) designed and conducted the Newhints study. All authors critically reviewed the manuscript, and approved the final manuscript as submitted.
